# Supplementary material for: Geopolitical species revisited: genomic and morphological data indicate that the roundtail chub Gila robusta species complex (Teleostei, Cyprinidae) is a single species
Source: PeerJ. 2018 Sep 27;6:e5605. doi: 10.7717/peerj.5605 (PMC6167970; doi:10.7717/peerj.5605)
Supplement: Supplemental Information 1 — Photo of note written by R.R. Miller in 1945 explaining error in catalog numbers of G. robusta reported by Jordan and Evermann 1896:227. This note is included with the type specimens of this species. [file peerj-06-5605-s001.pdf]

These, the types of *Gela robusta* B. & G., are cat. no. 246. They were reëntered by error as 47983 and attributed to nos. 276 & 277, which are cods! R. R. Miller
(over) III: 1945

Nos 276 & 277 were attributed as type nos. of this species, by error, by Jordan & Evermann, 1896: 227.
