# Supplementary material for: Geopolitical species revisited: genomic and morphological data indicate that the roundtail chub Gila robusta species complex (Teleostei, Cyprinidae) is a single species
Source: PeerJ. 2018 Sep 27;6:e5605. doi: 10.7717/peerj.5605 (PMC6167970; doi:10.7717/peerj.5605)
Supplement: Supplemental Information 2 [file peerj-06-5605-s002.docx]

**Supplementary Materials**

These analyses support the phylogenetic findings presented in the main text. However, because we designed the sampling for taxonomic and phlyogenetic, rather than population genetic analyses, we are hesitant about the sample size for these analyses, and include them as supporting information for those who wish to see these analyses and to accompany and support the remainder of the study.


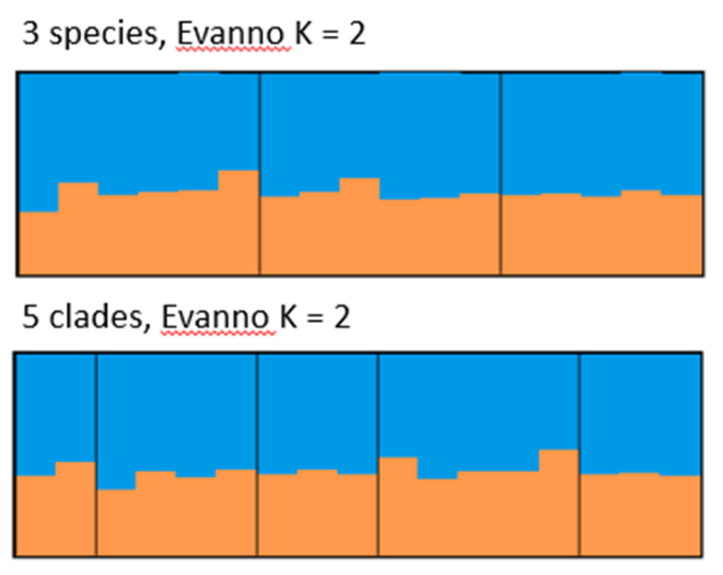


**Supplementary Figure 1:** Structure plots for the 3 members of the *Gila robusta* species complex, and an alternate 5 clades example. All permutations of the analysis returned K=2 with either informed or uninformed priors. Neither grouping is supported by the analysis which assigns all individuals with roughly equal proportions to 2 unknown groupings across all analyses, a common result when the true number of groups is actually one (Janes et al. 2017).


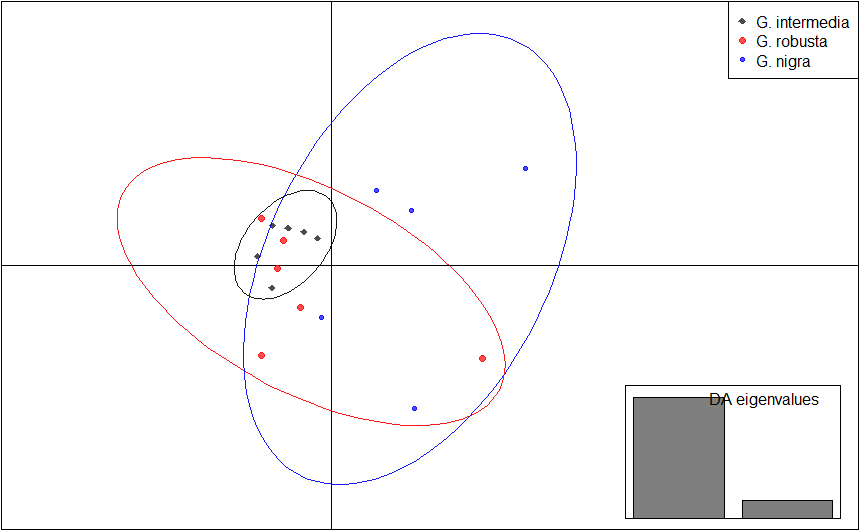


**Supplementary Figure 2:** Discriminate analysis of principle components (DAPC) implemented in R, for *Gila intermedia* (black), *G. robusta* (red) and *G. nigra* (blue). The ellipse of each respective color represents the 95% confidence interval for each group, and indicates broad overlap and no discriminatory power for the three nominal species.

**Methods**

STRUCTURE v.2.3.4 (Pritchard, Stephens & Donnelly, 2000) was run using a model assuming admixture and correlated allele frequencies, as we expect current gene flow and many shared alleles among morphotypes (Falush, Stephens & Pritchard, 2003). We ran analyses with both uninformed and informed priors for a three species model and five clade model. Values of K ranged from 1 to 17 with five replicate runs for each. Each analysis consisted of a burn-in of 500,000 steps followed by 10,000,000 MCMC iterations. We used delta K (Evanno, Regnaut & Goudet, 2005) from Structure Harvester v.0.6.94 to determine which K was the best fit to the data (Earl & vonHoldt, 2012). The 5 replicate runs were averaged using CLUMPP v.1.1.2 (Jakobsson & Rosenberg, 2007).

Discriminant analysis of principal components (DAPC) was implemented in the package ADEGENET v.2.1.1 (Jombart, 2008) for R v.3.5.1 (R core team).To determine the optimal number of principal components (PCs) to retain for the DAPC analysis, we used the cross-validation function for DAPC (Jombart, Devillard & Balloux, 2010). Due to the low within group sample size we used a training set of 0.5 to ensure that a minimum of 2 individuals were in each of the training and validation groups. The DAPC was run using 3 PCs and 2 discriminant functions.

**References**

Earl DA , vonHoldt BM. 2012. STRUCTURE HARVESTER: a website and program for visualizing STRUCTURE output and implementing the Evanno method. *Conservation Genetics Resources* **4**:359-361 doi: 10.1007/s12686-011-9548-7

Evanno G, Regnaut S, Goudet J. 2005. Detecting the number of clusters of individuals using the software structure: A simulation study. *Molecular Ecology* **14**:2611–2620. <https://doi.org/10.1111/j>. 1365-294X.2005.02553.x

Falush D, Stephens M, Pritchard JK. 2003. Inference of population structure using multilocus genotype data: Linked loci and correlated allele frequencies. *Genetics* **164**: 1567–1587.

Jakobsson M, Rosenberg NA. 2007. CLUMPP: A cluster matching and permutation program for dealing with label switching and multimodality in analysis of population structure. *Bioinformatics* 23:1801–1806.

Janes JK, Miller JM, Dupuis JR, Malenfant RM, Gorrell JC, Cullingham CI, Andrew RL. 2017. The K= 2 conundrum. *Molecular Ecology* 26(14):3594-3602.

Jombart T. 2008. adegenet: a R package for the multivariate analysis of genetic markers Bioinformatics 24:1403-1405. doi: 10.1093/bioinformatics/btn129

Jombart T, Devillard S, Balloux F. 2010. Discriminant analysis of principal components: a new method for the analysis of genetically structured populations. *BMC Genetics* **11**:94. doi:10.1186/1471-2156-11-94

Pritchard JK, Stephens M, Donnelly P. 2000. Inference of population structure using multilocus genotype data. *Genetics* **155**:945-959.
