# Supplementary material for: Geopolitical species revisited: genomic and morphological data indicate that the roundtail chub Gila robusta species complex (Teleostei, Cyprinidae) is a single species
Source: PeerJ. 2018 Sep 27;6:e5605. doi: 10.7717/peerj.5605 (PMC6167970; doi:10.7717/peerj.5605)
Supplement: Supplemental Information 3 [file peerj-06-5605-s003.docx]

|  | Linear Regression | | F-test | | T-test | |
| --- | --- | --- | --- | --- | --- | --- |
|  | R-squared | p-value | F | p-value | T | p-value |
| Greatest Body depth^1^ | 0.93 | 2.2e-16 | 0.98 | 0.99 | -1.01 | 0.84 |
| Head length^1^ | 0.99 | 2.2e-16 | 1.05 | 0.90 | -2.9 | 0.99 |
| Head width^4^ | 0.95 | 2.2e-16 | 0.41 | 0.13 | -3.04 | 0.99 |
| Head depth^4^ | 0.97 | 2.2e-16 | 0.66 | 0.49 | -1.5 | 0.93 |
| Snout length^2^ | 0.97 | 2.2e-16 | 2.06 | 0.19 | -0.08 | 0.53 |
| Mandible length^2^ | 0.97 | 2.2e-16 | 2.52 | 0.09 | -1.06 | 0.15 |
| Orbit diameter^2^ | 0.79 | 7.7e-11 | 3.42 | 0.03 | -0.34 | 0.37 |
| Interorbital width^2^ | 0.98 | 2.2e-16 | 1.41 | 0.52 | -1.8 | 0.96 |
| Upper-jaw length^2^ | 0.88 | 1.2e-14 | 1.4 | 0.54 | 1.01 | 0.84 |
| Caudal-peduncle depth^4^ | 0.87 | 2.6e-14 | 0.51 | 0.26 | -0.96 | 0.83 |
| Caudal peduncle length^1^ | 0.88 | 1.2e-14 | 2.63 | 0.08 | -0.67 | 0.25 |
| Predorsal length^1^ | 0.98 | 2.2e-16 | 1.789 | 0.28 | -3.06 | 0.99 |
| Preanal length^1^ | 0.99 | 2.2e-16 | 1.01 | 0.96 | -1.53 | 0.93 |
| Pectoral insertion to pelvic insertion^1^ | 0.73 | 7.3e-10 | 26.19 | 7.68E-08 | -1.46 | 0.91 |
| Anal to Caudal length^1^ | 0.96 | 2.2e-16 | 0.83 | 0.77 | -0.66 | 0.53 |
| Origin of anal fin to hypural plate^1^ | 0.95 | 2.2e-16 | 0.11 | 0.001 | -2.06 | 0.97 |
| Prepelvic length^1^ | 0.96 | 2.2e-16 | 0.18 | 0.01 | -1.74 | 0.95 |
| Pectoral-fin length^1^ | 0.82 | 2.6e-12 | 0.98 | 0.99 | -2.26 | 0.98 |
| Anal fin length^1^ | 0.86 | 2.0e-13 | 2.16 | 0.16 | -1.25 | 0.89 |
| Pelvic-fin length^1^ | 0.92 | 2.2e-16 | 1.13 | 0.80 | -2.56 | 0.99 |
| Dorsal fin length^1^ | 0.92 | 2.5e-15 | 2.37 | 0.13 | -0.24 | 0.59 |
