# Supplementary material for: Geopolitical species revisited: genomic and morphological data indicate that the roundtail chub Gila robusta species complex (Teleostei, Cyprinidae) is a single species
Source: PeerJ. 2018 Sep 27;6:e5605. doi: 10.7717/peerj.5605 (PMC6167970; doi:10.7717/peerj.5605)
Supplement: Supplemental Information 4 [file peerj-06-5605-s004.docx]

|  | ABBA | BABA | P-Value | D | D (Jackknifed) | Fd(P2, P3) | Fd(P2, P3) (Jackknifed) | Fd(P1, P3) | Fd(P1, P3) (Jackknifed) | Z score for D | Z score for Fd(P2, P3) | Z score for Fd(P1, P3) |
| --- | --- | --- | --- | --- | --- | --- | --- | --- | --- | --- | --- | --- |
| ((100,105)HBC) | 23 | 10 | 0.043 | 0.394 | 0.394 | 0.002 | 0.002 | 0.000 | 0.000 | 4.596 | 0.029 | -0.025 |
| ((100,150)HBC) | 23 | 9 | 0.024 | 0.438 | 0.438 | 0.003 | 0.003 | 0.000 | 0.000 | 3.669 | 0.024 | -0.017 |
| ((100,152)HBC) | 14 | 209 | 0.000 | -0.874 | -0.874 | 0.000 | 0.074 | 0.037 | 0.000 | -3.694 | 0.313 | -0.452 |
| ((100,141)HBC) | 22 | 9 | 0.035 | 0.419 | 0.419 | 0.002 | 0.002 | 0.000 | 0.000 | 3.856 | 0.021 | -0.020 |
| ((100,125)HBC) | 343 | 8 | 0.000 | 0.954 | 0.954 | 0.068 | 0.039 | 0.000 | 0.000 | 9.473 | 0.386 | -0.570 |
| ((100,132)HBC) | 322 | 10 | 0.000 | 0.940 | 0.940 | 0.054 | 0.042 | 0.000 | 0.000 | 6.566 | 0.295 | -0.381 |
| ((122,105)HBC) | 15 | 334 | 0.000 | -0.914 | -0.914 | 0.000 | 0.000 | 0.055 | 0.042 | -4.269 | -0.174 | 0.194 |
| ((133,150)HBC) | 15 | 333 | 0.000 | -0.914 | -0.914 | 0.000 | 0.000 | 0.048 | 0.038 | -4.263 | -0.191 | 0.178 |
| ((133,152)HBC) | 14 | 541 | 0.000 | -0.950 | -0.950 | 0.000 | 0.000 | 0.083 | 0.073 | -75.053 | -5.685 | 5.757 |
| ((133,112)HBC) | 11 | 332 | 0.000 | -0.936 | -0.936 | 0.000 | 0.000 | 0.054 | 0.041 | -3.198 | -0.233 | 0.141 |
| ((133,162)HBC) | 15 | 630 | 0.000 | -0.953 | -0.953 | 0.000 | 0.000 | 0.112 | 0.091 | -943.509 | -97.475 | 89.958 |
| ((133,141)HBC) | 14 | 333 | 0.000 | -0.919 | -0.919 | 0.000 | 0.000 | 0.057 | 0.048 | -4.262 | -0.207 | 0.222 |
| ((133,122)HBC) | 36 | 852 | 0.000 | -0.919 | -0.919 | 0.000 | 0.000 | 0.171 | 0.127 | -166.911 | -48.863 | 23.046 |
| ((133,145)HBC) | 36 | 860 | 0.000 | -0.920 | -0.920 | 0.000 | 0.000 | 0.187 | 0.152 | -208.762 | -25.486 | 34.575 |
| ((133,GR1)HBC) | 8 | 334 | 0.000 | -0.953 | -0.953 | 0.000 | 0.000 | 0.042 | 0.032 | -4.507 | -0.273 | 0.151 |
| ((133,155)HBC) | 7 | 336 | 0.000 | -0.959 | -0.959 | 0.000 | 0.000 | 0.055 | 0.043 | -5.478 | -0.381 | 0.248 |
| ((133,140)HBC) | 9 | 334 | 0.000 | -0.948 | -0.948 | 0.000 | 0.000 | 0.055 | 0.042 | -4.052 | -0.204 | 0.181 |
| ((133,121)HBC) | 8 | 333 | 0.000 | -0.953 | -0.953 | 0.000 | 0.000 | 0.055 | 0.042 | -4.259 | -0.213 | 0.187 |
| ((105,162)HBC) | 4 | 300 | 0.000 | -0.974 | -0.974 | 0.000 | 0.195 | 0.058 | 0.000 | -8.587 | 1.720 | -0.697 |
| ((105,125)HBC) | 332 | 10 | 0.000 | 0.942 | 0.942 | 0.063 | 0.036 | 0.000 | 0.000 | 9.192 | 0.348 | -0.364 |
| ((105,155)HBC) | 4 | 14 | 0.046 | -0.556 | -0.556 | 0.000 | 0.000 | 0.002 | 0.002 | -11.180 | -0.033 | 0.034 |
| ((105,132)HBC) | 312 | 13 | 0.000 | 0.920 | 0.920 | 0.051 | 0.039 | 0.000 | 0.000 | 5.612 | 0.240 | -0.221 |
| ((150,125)HBC) | 331 | 10 | 0.000 | 0.941 | 0.941 | 0.061 | 0.045 | 0.000 | 0.000 | 9.179 | 0.437 | -0.531 |
| ((150,155)HBC) | 3 | 14 | 0.019 | -0.647 | -0.647 | 0.000 | 0.000 | 0.002 | 0.002 | -4.667 | -0.011 | 0.015 |
| ((150,132)HBC) | 311 | 13 | 0.000 | 0.920 | 0.920 | 0.046 | 0.037 | 0.000 | 0.000 | 5.604 | 0.224 | -0.240 |
| ((152,141)HBC) | 211 | 3 | 0.000 | 0.972 | 0.972 | 0.037 | 0.000 | 0.000 | 0.075 | 165.450 | -5.980 | 12.760 |
| ((152,122)HBC) | 211 | 9 | 0.000 | 0.918 | 0.918 | 0.044 | 0.000 | 0.000 | 0.043 | 4.278 | -0.430 | 0.199 |
| ((152,GR1)HBC) | 212 | 11 | 0.000 | 0.901 | 0.901 | 0.040 | 0.000 | 0.000 | 0.057 | 3.583 | -0.388 | 0.227 |
| ((152,125)HBC) | 539 | 9 | 0.000 | 0.967 | 0.967 | 0.100 | 0.099 | 0.000 | 0.000 | 95.937 | 9.869 | -7.196 |
| ((152,155)HBC) | 211 | 13 | 0.000 | 0.884 | 0.884 | 0.045 | 0.000 | 0.000 | 0.074 | 4.360 | -0.409 | 0.365 |
| ((152,140)HBC) | 214 | 12 | 0.000 | 0.894 | 0.894 | 0.042 | 0.000 | 0.000 | 0.074 | 4.369 | -0.216 | 0.363 |
| ((152,132)HBC) | 512 | 5 | 0.000 | 0.981 | 0.981 | 0.086 | 0.076 | 0.000 | 0.000 | 41.764 | 3.219 | -3.012 |
| ((152,121)HBC) | 213 | 11 | 0.000 | 0.902 | 0.902 | 0.041 | 0.000 | 0.000 | 0.075 | 4.338 | -0.229 | 0.358 |
| ((112,162)HBC) | 9 | 303 | 0.000 | -0.942 | -0.942 | 0.000 | 0.130 | 0.077 | 0.000 | -3.179 | 0.437 | -0.364 |
| ((112,125)HBC) | 330 | 6 | 0.000 | 0.964 | 0.964 | 0.066 | 0.035 | 0.000 | 0.000 | 4.385 | 0.161 | -0.308 |
| ((112,132)HBC) | 309 | 8 | 0.000 | 0.950 | 0.950 | 0.052 | 0.039 | 0.000 | 0.000 | 3.898 | 0.161 | -0.266 |
| ((162,122)HBC) | 300 | 10 | 0.000 | 0.935 | 0.935 | 0.065 | 0.000 | 0.000 | 0.072 | 3.158 | -0.435 | 0.243 |
| ((162,125)HBC) | 628 | 10 | 0.000 | 0.969 | 0.969 | 0.119 | 0.112 | 0.000 | 0.000 | 56.755 | 6.583 | -4.257 |
| ((162,155)HBC) | 300 | 14 | 0.000 | 0.911 | 0.911 | 0.067 | 0.000 | 0.000 | 0.126 | 3.140 | -0.401 | 0.433 |
| ((162,140)HBC) | 302 | 12 | 0.000 | 0.924 | 0.924 | 0.059 | 0.000 | 0.000 | 0.127 | 3.160 | -0.235 | 0.435 |
| ((162,132)HBC) | 607 | 12 | 0.000 | 0.961 | 0.961 | 0.110 | 0.087 | 0.000 | 0.000 | 77.759 | 7.003 | -6.961 |
| ((162,121)HBC) | 301 | 11 | 0.000 | 0.929 | 0.929 | 0.058 | 0.000 | 0.000 | 0.127 | 3.159 | -0.247 | 0.432 |
| ((141,125)HBC) | 331 | 9 | 0.000 | 0.947 | 0.947 | 0.071 | 0.046 | 0.000 | 0.000 | 9.165 | 0.443 | -0.431 |
| ((141,155)HBC) | 3 | 13 | 0.032 | -0.625 | -0.625 | 0.000 | 0.000 | 0.002 | 0.001 | -5.000 | -0.013 | 0.012 |
| ((141,132)HBC) | 303 | 4 | 0.000 | 0.974 | 0.974 | 0.056 | 0.041 | 0.000 | 0.000 | 6.114 | 0.258 | -0.219 |
| ((122,132)HBC) | 310 | 5 | 0.000 | 0.968 | 0.968 | 0.052 | 0.032 | 0.000 | 0.000 | 6.195 | 0.205 | -0.405 |
| ((GR1,132)HBC) | 312 | 6 | 0.000 | 0.962 | 0.962 | 0.040 | 0.031 | 0.000 | 0.000 | 6.225 | 0.199 | -0.356 |
| ((125,140)HBC) | 5 | 333 | 0.000 | -0.970 | -0.970 | 0.000 | 0.000 | 0.067 | 0.037 | -6.423 | -0.317 | 0.248 |
| ((125,121)HBC) | 4 | 332 | 0.000 | -0.976 | -0.976 | 0.000 | 0.000 | 0.069 | 0.036 | -7.416 | -0.364 | 0.277 |
| ((155,132)HBC) | 314 | 5 | 0.000 | 0.097 | 0.097 | 0.053 | 0.042 | 0.000 | 0.000 | 7.712 | 0.335 | -0.510 |
